# Advanced X-ray imaging at beamline 07 of the SAGA Light Source

**DOI:** 10.1107/S1600577521009553

**Published:** 2021-10-21

**Authors:** Akio Yoneyama, Satoshi Takeya, Thet Thet Lwin, Daiko Takamatsu, Rika Baba, Kumiko Konishi, Ryusei Fujita, Keisuke Kobayashi, Akio Shima, Masahide Kawamoto, Hiroyuki Setoyama, Kotaro Ishiji, Yoshiki Seno

**Affiliations:** a Beamline Group, SAGA Light Source, 8-7 Yayoigaoka, Tosu, Saga 841-0005, Japan; b Allied Health Sciences, Kitasato University, 1-15-1 Kitasato, Minamiku, Sagamihara, Kanagawa 252-0373, Japan; c Research and Development Group, Hitachi Ltd, 1-280 Higashi-koigakubo, Kokubunji, Tokyo 185-8601, Japan; d National Metrology Institute of Japan, National Institute of Advanced Industrial Science and Technology (AIST), Central 5, 1-1-1 Higashi, Tukuba, Ibaraki 305-8565, Japan

**Keywords:** micro-CT, phase-contrast CT, low-temperature CT, time-resolved topography, Ge double-crystal monochromator, operando topography

## Abstract

The SAGA Light Source provides X-ray imaging resources based on high-intensity synchrotron radiation (SR) emitted from the superconducting wiggler at beamline 07 (BL07). By combining quasi-monochromatic SR obtained by the newly installed water-cooled metal filter and monochromatic SR selected by a Ge double-crystal monochromator (DCM) with high-resolution lens-coupled X-ray imagers, fast and low-dose micro-computed tomography (CT), fast phase-contrast CT using grating-based X-ray interferometry, and 2D micro-X-ray absorption fine structure analysis can be performed. In addition, by combining monochromatic SR obtained by a Si DCM with large-area fiber-coupled X-ray imagers, high-sensitivity phase-contrast CT using crystal-based X-ray interferometry can be performed. Low-temperature CT can be performed using the newly installed cryogenic system, and time-resolved analysis of the crystallinity of semiconductor devices in operation can be performed using a time-resolved topography system. The details of each instrument and imaging method, together with exemplary measurements, are presented.

## Abstract 2

The paper describes the new X-ray imaging resources, including micro-computed tomography (CT), phase-contrast CT, low-temperature CT and time-resolved topography, that are available at beamline 07 of the SAGA Light Source.

## 1. Introduction

Synchrotron radiation (SR) is several orders of magnitude brighter than X-rays from conventional X-ray tubes, and its high brilliance has been used for X-ray imaging in various fields such as biomedicine, materials science, archeology and geochemistry. The SAGA Light Source facility (stored energy 1.4 GeV, maximum ring current 300 mA) in Saga Prefecture, Japan, was established in 2006 as a source of SR dedicated to a wide range of experiments. SR from a bending magnet (BL15) and from a superconducting wiggler (BL07) (Sumitani *et al.*, 2013 ▸) has been used to perform computed tomography (CT) and diffraction-enhanced phase-contrast CT experiments. X-ray fluorescence microscopy using an X-ray beam focused by a Fresnel zone plate (FZP) has also been conducted as well (Sumitani *et al.*, 2011 ▸). Furthermore, imaging methods such as the sub-pixel-shift method that uses photon-counting detectors to improve the spatial resolution (Yoneyama, Baba, Sumitani *et al.*, 2015 ▸) and dual-energy CT to calculate the effective atomic number have been developed for use at the site. X-ray topography equipment using X-ray film has also been made available (Ishiji *et al.*, 2011 ▸).

We have been developing advanced X-ray imaging techniques at BL07 to enable researchers to conduct non-destructive investigations. In particular, we have developed a fast phase-contrast CT system using diffraction-enhanced imaging. The system has been used to make 3D observations within a 10 min scanning time (Yoneyama *et al.*, 2020 ▸). In addition, we have developed a scanning X-ray fluorescence microscopy (SXFM) system using total-reflection mirrors and white SR. This system has been used to map the distributions of elements such as S, Cl, K and Ca in plant seeds (Yoneyama & Kawamoto, 2020 ▸). We have also developed high-resolution lens-coupled X-ray imagers using optimized phosphor (Yoneyama *et al.*, 2021 ▸). Most recently, we developed a water-cooled metal filter, a Ge double-crystal monochromator (DCM), phase-contrast CT systems using a grating-based and a crystal-based X-ray interferometer, a cold-nitro­gen-gas cryogenic system and a time-resolved X-ray topography system.

As a result of these developments, the following forms of X-ray imaging can now be performed at the SAGA Light Source: (i) fast high-spatial-resolution CT (fast micro-CT) using high-intensity quasi-monochromatic (pink) SR, (ii) low-dose micro-CT, (iii) micro-2D X-ray absorption fine structure (XAFS) analysis, (iv) fast phase-contrast CT, (v) high-sensitivity phase-contrast CT, (vi) low-temperature CT and (vii) time-resolved analysis of crystallinity of semiconductor devices in operation. In this article, we report on the experimental instruments and imaging methods, as well as some exemplary applications of the advanced X-ray imaging methods carried out at BL07 of the SAGA Light Source.

## 2. Beamline layout, experimental instrumentation and SR properties

### 2.1. Outline

**Figure 1:**
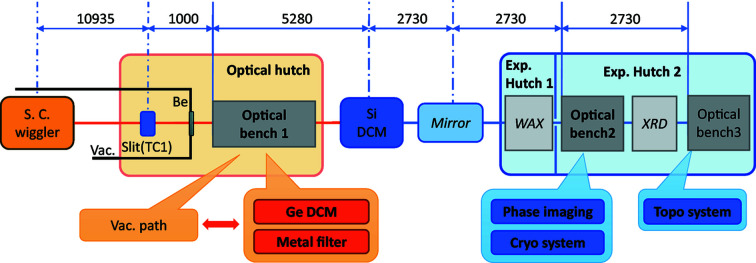
Layout of BL07 at SAGA Light Source and newly installed instrumentation (bold).

Beamline 07 (BL07) of the SAGA Light Source consists mainly of a superconducting magnet wiggler, an optical hutch, a Si DCM (Sumitani *et al.*, 2013 ▸), a total-reflection focusing mirror, and experimental hutches 1 and 2, as shown in Fig. 1 ▸ (Kawamoto *et al.*, 2010 ▸). White SR emitted from the wiggler and shaped by a slit consisting of water-cooled Ta blades can be used directly in the optical hutch. Parallel SR monochromated by the Si DCM using Si (220) or Si (111) diffraction can be employed in experimental hutches 1 and 2. In addition, by using the focusing mirror, focused monochromatic SR without higher-order effects, which is especially useful for XAFS measurements, can be used in the experimental hutches. Note that a vacuum path has been installed in the optical hutch to reduce flux loss caused by X-ray scattering in the air when SR after passing the Si DCM is used.

The optical hutch has been used to perform white SR irradiation experiments on plant seeds (Sakamoto *et al.*, 2019 ▸). In particular, SR irradiation was used to genetically modify various flowers that blossomed in unnatural colors. Experimental hutch 1 has wide-angle X-ray diffraction (WAXD) and wide-angle X-ray scattering (WAXS) (Ohtani *et al.*, 2017 ▸) equipment. It has been used to perform protein crystallography (PX) (Arai *et al.*, 2015 ▸) measurements. Experimental hutch 2 has a six-axis diffractometer and an XAFS measurement system which are installed in a tandem arrangement, and it has been used to perform X-ray diffraction (XRD), XAFS (Okajima *et al.*, 2018 ▸) and X-ray fluorescence analysis (XRF) (Tabata & Ueda, 2017 ▸) experiments using monochromatic SR above 10 keV.

### 2.2. Upgrade of X-ray optics

#### 2.2.1. Compact DCM using Ge (111) diffraction

**Figure 2:**
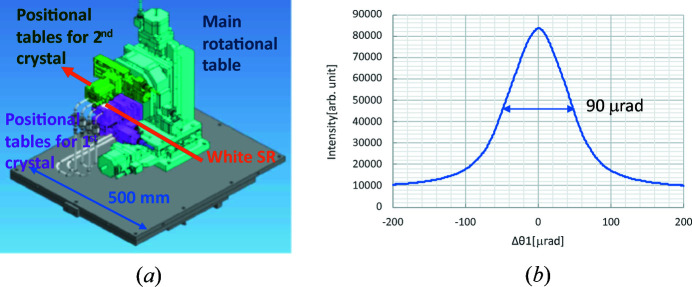
(*a*) Schematic view of the compact Ge DCM and (*b*) rocking curve obtained by rotating the first crystal.

A portable compact DCM using Ge (111) diffraction was developed and installed in the optical hutch. As shown in Fig. 2 ▸(*a*), the compact Ge DCM consists of the first crystal and its positioning tables (purple), the second crystal and its positioning tables (green), and the main rotational mount of the crystal-positioning tables and its positioning tables (light blue). The main rotational-axis angle (Bragg angle) ranges from 5 to 18.5°, corresponding to 7 to 20 keV. The beam size is 20 mm × 2 mm, which is large enough for micro-CT and micro-XAFS. Since the compact DCM must be removed from the SR path in order to use the downstream experimental hutches, it uses small Ge crystals (30 mm × 35 mm), which reduce its length to less than 500 mm for easy transport. Moreover, the main chamber of the DCM substitutes He gas for a vacuum, and the chamber itself is made of acrylic to reduce its weight (20 kg). Owing to the short distance from the wiggler (∼12 m) and the wide bandwidth (diffraction width) of Ge (111), high-intensity monochromatic SR can be used.

Fig. 2 ▸(*b*) shows a rocking curve obtained by rotating the first crystal. The full width at half-maximum (FWHM) is 90 µrad, which is consistent with the FWHM calculated from the Ge diffraction width and the vertical beam divergence of the SR.

#### 2.2.2. Water-cooled metal filter

A water-cooled metal filter is installed in the optical hutch to narrow the energy band and optimize the peak energy of white SR depending on the samples’ thickness and composition. A combination of metal filters such as aluminium, copper, nickel and zirconium of 0.1, 0.2 and 0.5 mm thickness can be chosen.

### 2.3. SR flux

**Figure 3:**
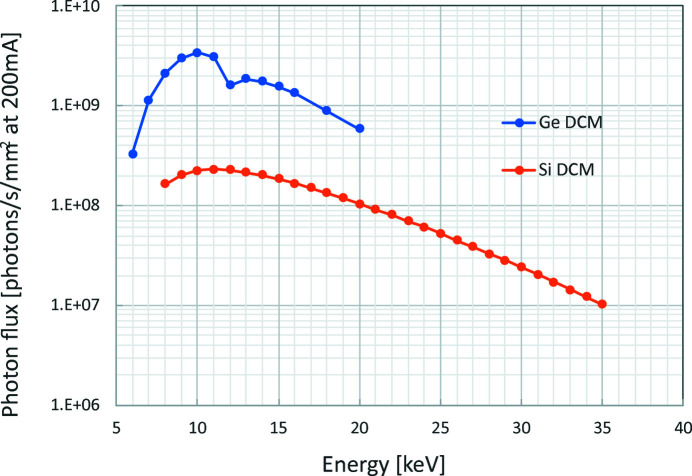
Measured X-ray photon flux (photons s^−1^ mm^−2^) at BL07 of SAGA Light Source downstream of Ge and Si DCM.

The photon fluxes of the monochromatic SR obtained by the Ge and Si DCMs are shown in Fig. 3 ▸. The flux was calculated from the detected ionization current of the ion chamber (OKEN S-1329) and the gas composition (50% N_2_ and 50% Ar) by using *Hephaestus* software (Ravel & Newville, 2005 ▸). Since the top-up operation is not supported at the SAGA Light Source and, hence, the photon flux decreases with time, the flux was normalized at a ring current of 200 mA, which is 2/3 of the maximum stored current. Moreover, a four-blade slit (*Q* slit) with a 1 mm × 1 mm opening was set upstream of the ion chamber to normalize the beam size.

The results in the figure show that the photon flux using the Ge DCM amounts to 2 × 10^9^ photons s^−1^ mm^−2^ at 10 keV, which is about 10× bigger than that of the Si DCM. The peak energy is 10 keV, which is slightly higher than that of the white SR (Kawamoto *et al.*, 2010 ▸) due to the absorption of lower-energy SR by the Be window and the air between that window and the ion chamber. The *K* edge of Ge is 11.1 keV; therefore, the flux at 12 keV is reduced by about 10%.

**Figure 4:**
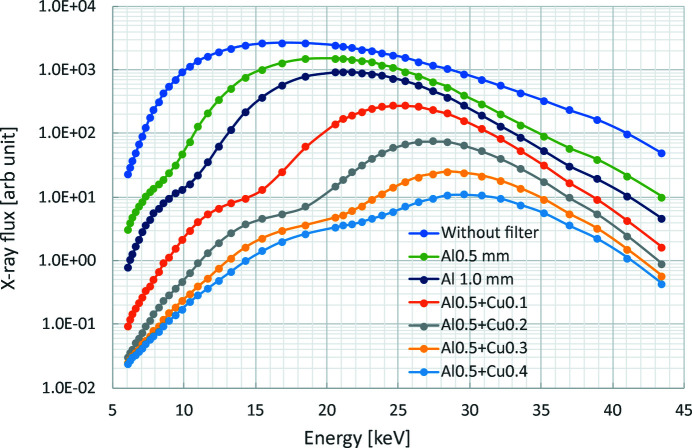
Spectra of quasi-monochromatic SR obtained using various metal filters. Peak energy can be varied by changing the type and thickness of the metal filter.

Fig. 4 ▸ shows the SR spectra as a result of adding different metal filters in the beam path. The spectral data were obtained by θ–2θ scanning of a Si single crystal by an X-ray diffractometer placed on the optical bench of the optical hutch [details of the measurements can be found in a previous report (Yoneyama & Kawamoto, 2020 ▸)]. This result shows that the peak energy can be shifted by changing the metal filter’s type and thickness; in particular, it can be set to 15 keV by using 1 mm-thick aluminium and 30 keV by using 0.5 mm-thick copper. Note that we can use all of the SR energy for investigations, whereby the photon flux is about 1000× higher than that of monochromatic SR.

### 2.4. X-ray imager

Two custom-made microscopic X-ray imagers (Kenvy 1 and 2) and two commercial fiber-coupled X-ray imagers with different fields of view are available at the SAGA Light Source. The main specifications of each imager are listed in Table 1 ▸.

#### 2.4.1. Microscopic X-ray imagers (Kenvy 1 and Kenvy 2)

The microscopic X-ray imagers, Kenvy 1 for white SR (Yoneyama *et al.*, 2016 ▸) and Kenvy 2 for monochromatic SR (Yoneyama *et al.*, 2021 ▸), each consist of a phosphor that converts X-rays into visible light, a lens system that transfers the converted visible light to a visible-light camera and an sCMOS visible-light camera (Koch *et al.*, 1998 ▸). The lens system employs an infinity optical system, and the magnification can be changed by exchanging the objective lens. Kenvy 1 has a prism in the middle of the lens system; only visible light is reflected 90° upward to protect the sCMOS camera from being damaged by white SR. The standard phosphor is Lu_3_Al_5_O_12_:Ce (LuAG) for Kenvy 1 and CsI:Tl (CsI) for Kenvy 2. sCMOS cameras (Andor NEO 5.5 and Zyla 4.2) are used to capture the visible light. The pixel size is 6.5 µm, while the effective pixel sizes are 1.3 and 0.65 µm using ×5 and ×10 objective lenses, respectively.

#### 2.4.2. Fiber-coupled X-ray imagers

The VHR’s fiber optics have a taper ratio of 1:1.5, the pixel size is 12.5 µm, the pixel number is 4008 × 2650, the field of view is 50 mm × 35 mm, and the image transfer rate is 1.6 frame s^−1^ for a full image. A 30 µm-thick Gd_2_O_2_S:Tb (GOS) phosphor is used to convert the SR into visible light. The Zyla’s fiber optics have a taper ratio of 1:1, the pixel size is 6.5 µm, the pixel number is 2560 × 2180, the field of view is 16 mm × 13 mm, and the transfer rate is 100 frame s^−1^ for a full image. A 100 µm-thick CsI phosphor is used.

## 3. Advanced X-ray imaging

### 3.1. Imaging methods

**Figure 5:**
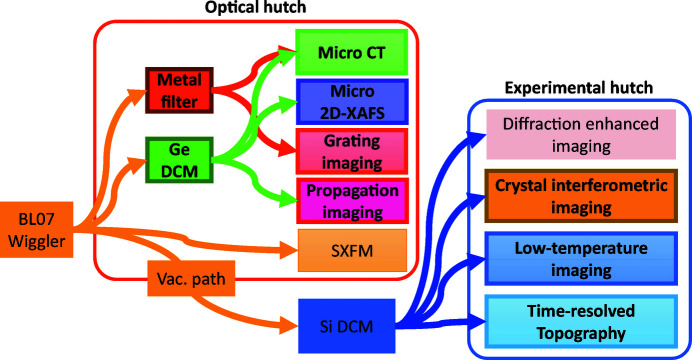
Chart of imaging methods at BL07. Colored arrows indicate the X-ray paths.

A chart of the imaging methods available at BL07 is shown in Fig. 5 ▸, and the detailed specifications and typical applications of each imaging method are listed in Table 2 ▸. Although the SAGA Light Source is a synchrotron facility with a medium ring energy, various imaging methods are possible by combining the white, quasi-monochromatic and monochromatic SR with the various X-ray imagers described above. Details of the imaging methods (indicated by the framed boxes) enabled by the newly installed instruments and exemplary results are described below.

### 3.2. Micro-CT

Fast micro-CT using a combination of Kenvy 1 and quasi-monochromatic SR obtained by the metal filter and low-dose micro-CT using a combination of Kenvy 2 and monochromatic SR selected by the Ge DCM (shown as the green boxes in Fig. 5 ▸) can be performed in the optical hutch. Both micro-CTs have a parallel-beam geometry in which the propagation distance (PD: the distance between the sample and the X-ray imager) is less than 5 mm to suppress penumbral blurring. The main advantages are a wide field of view and the ability to use high-flux SR without any intensity loss from X-ray optical devices.

#### 3.2.1. Fast micro-CT using quasi-monochromatic SR

**Figure 6:**
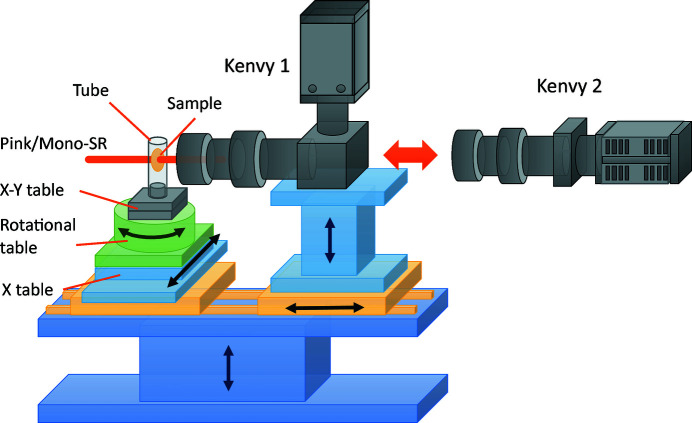
Schematic view of the fast and low-dose micro-CT system. Kenvy 1, for fast micro-CT, and Kenvy 2, for low-dose micro-CT, are exchangeable.

The fast micro-CT system consists of a sample-positioning system and Kenvy 1, as shown in Fig. 6 ▸. Both are mounted on a large *Z* table to adjust the heights relative to the SR optical path simultaneously. The sample-positioning system consists of a large *X* table and rotational table driven by stepping motors and a small manual *X*–*Y* table to adjust the sample center to the rotational center. The sample usually is sealed in a 2 mm-diameter polyethyl­ene tube and attached to the sample holder. The PD is adjusted manually by moving Kenvy 1 along an optical rail.

**Figure 7:**
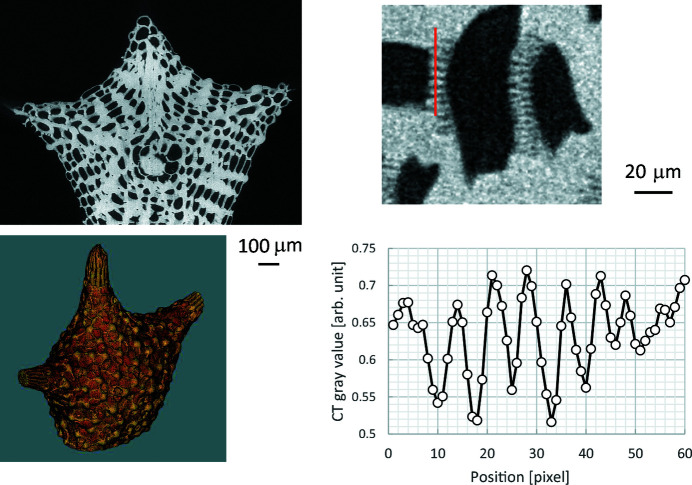
Images of a microfossil. Top left: cross-sectional image; bottom left: 3D volume rendering image; top right: magnified cross-sectional image; bottom right: line profile.

Fig. 7 ▸ shows an observation of a microfossil (benthic foraminifera, *Baculogypsina* sp.). The exposure time for each image was 100 ms, the number of projections was 2000 over 360° and the total measurement time was 200 s. Cu (0.1 mm) and Al (1.5 mm) were used as metal filters, and the peak energy was optimized to 25 keV (see Fig. 4 ▸). Owing to the optimized SR energy and good signal-to-noise ratio due to the high intensity of the quasi-monochromatic SR at the selected mean photon energy, not only the surface but also the internal microstructure is depicted with micrometre resolution. The pixel size of the magnified image in the top right and the line profile in the bottom right is 0.65 µm. As can be seen, fine structures are resolved to 3 pixels; the spatial resolution is estimated to be less than 2 µm (∼0.65 µm × 3). The total radiation dose of this observation was calculated to be about 3 MGy.

#### 3.2.2. Low-dose micro-CT using monochromatic SR

**Figure 8:**
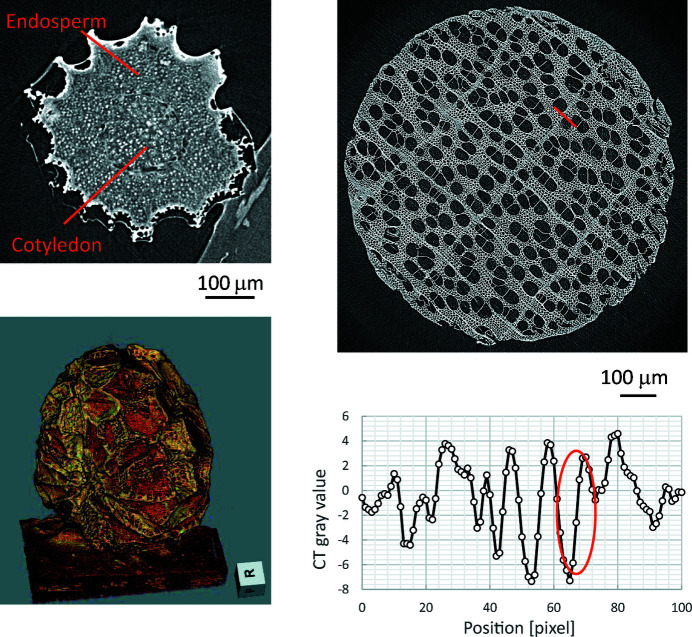
Images of *Eustoma grandiflorum* seed (top left: cross-sectional image; bottom left: 3D volume rendering image) and white birch (top right: cross-sectional image; bottom right: line profile) from low-dose micro-CT using monochromatic SR.

Low-dose micro-CT with minimized radiation damage and beam hardening can be performed using the same system, by exchanging Kenvy 1 with Kenvy 2 and the metal filter with the Ge DCM. This micro-CT is suitable for observing samples vulnerable to radiation damage, such as biomedical tissues and organic material. Fig. 8 ▸ shows imaging of a *Eustoma grandiflorum* seed and white birch. The SR energy was set to 9 keV, the exposure time for each image was set to 2 s, and the number of projections was 1000 and 2000 images over 360° for seed and birch, respectively. The magnification of the objective lens of Kenvy 2 is ×5, so the pixel size is 1.3 µm.

The results clearly show not only the detailed irregularities in the structure of the seed coat, but also the endosperm and the inside of the cotyledon. The CT gray value of the double-sided tape substrate (paper) is 0.48, and the fluctuation (standard deviation) of the background region is 0.02; therefore, the density resolution is estimated to be 30 mg cm^−3^, assuming that the paper’s density (recycled paper) is 0.80 g cm^−3^. In addition, the CT gray value changes from minimal to maximal within 3 pixels in the orange circle of the line profile (bottom right), so the spatial resolution is estimated to be less than 4 µm (3 × 1.3 µm).

#### 3.2.3. Micro-XAFS

**Figure 9:**
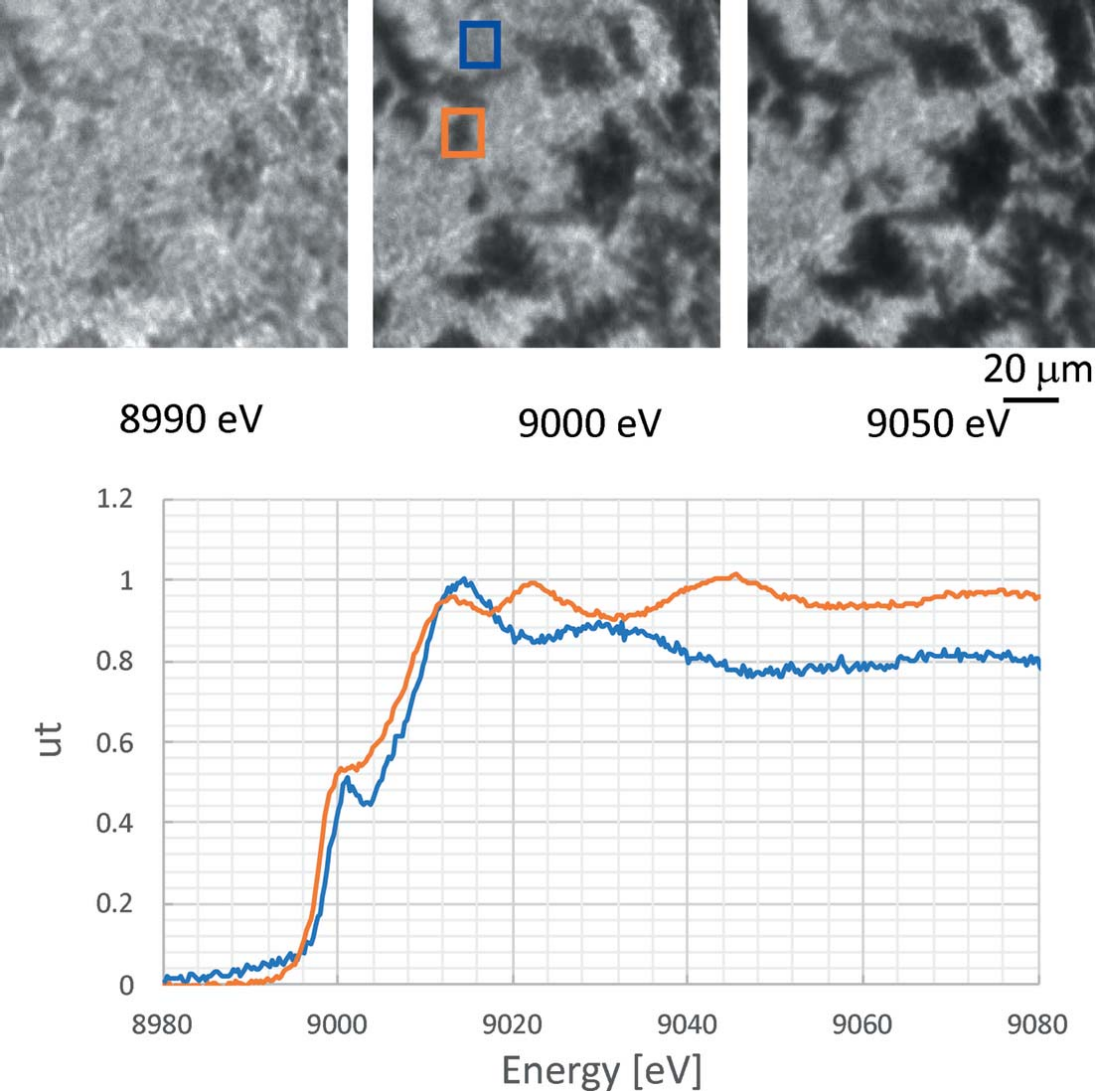
Micro-XAFS measurement results for a mixture of Cu and CuO_2_ powders at the Cu *K* edge. Top: projection images before Cu *K* edge (left), at Cu pre-edge (center) and after Cu *K* edge (right). Bottom: XAFS spectrum of blue and orange area indicated in top-center image.

Chemical state mappings made from XAFS measurements with micrometre-order spatial resolution (micro-XAFS) can be performed by combining SR monochromated by the Ge DCM with Kenvy 2 (shown as the purple box in Fig. 5 ▸). Fig. 9 ▸ shows the results of micro-XAFS measurements on a mixture of Cu and CuO_2_ powders near the Cu *K* edge. Each projection image was obtained by scanning the X-ray energy from 8.89 to 9.08 keV in 0.29 eV increments (12.2 to 11.8° in 0.0004° steps of the Ge DCM). The projection images (130 µm × 130 µm) at the top were obtained before the *K* edge (8990 eV) (left), at the pre-edge (9000 eV) (center) and after the *K* edge (9050 eV) (right). The spectra at the bottom are logarithms of the average transmitted X-ray intensity at each energy in the blue and orange regions (14 × 14 pixels) in the center image. The exposure time for obtaining each projection image was 2 s and the total measurement time was 30 min. The magnification of the objective lens of Kenvy 2 was ×10, so that the pixel size is 0.65 µm.

The spectrum in the blue area shows a strong white-line peak characteristic of oxides and a clear pre-edge, indicating that CuO_2_ is the main component. The orange-area spectrum has the same shape as that of the standard Cu foil, indicating that Cu is the main component. The size of each area is 9 µm × 9 µm (0.65 µm × 14 pixels), so the results show that the micro-XAFS system can measure the spatial distribution of chemical states with a spatial resolution of 9 µm.

### 3.3. Phase-contrast X-ray imaging (phase imaging)

Phase-contrast X-ray imaging (phase imaging), which uses the phase shift experienced by X-rays passing through a sample, is a powerful tool for non-destructive 3D observations. Since the cross section of the phase shift for light elements such as carbon, oxygen and nitro­gen in the hard X-ray region is more than 1000× larger than that of absorption (Momose & Fukuda, 1995 ▸; Momose *et al.*, 1996 ▸), fine observations can be performed even of biological soft tissues and organic materials, which are mainly composed of light elements. Four kinds of phase imaging for a large field of view can be classified according to the phase-detection method (Bonse & Busch, 1996 ▸; Bravin *et al.*, 2013 ▸; Momose, 2020 ▸): (i) grating-based (Talbot) interferometry using X-ray gratings (David *et al.*, 2002 ▸; Momose *et al.*, 2003 ▸; Weitkamp *et al.*, 2005 ▸; Pfeiffer *et al.*, 2006 ▸; Yashiro *et al.*, 2010 ▸), (ii) crystal-based X-ray interferometry (Momose, 1995 ▸; Momose *et al.*, 2001 ▸) using a crystal X-ray interferometer (Bonse & Hart, 1965 ▸), (iii) diffraction-enhanced imaging (DEI) using X-ray diffraction of a single crystal (Davis *et al.*, 1995 ▸; Chapman *et al.*, 1997 ▸) and (iv) propagation-based phase-contrast imaging using Fresnel diffraction (Snigirev *et al.*, 1995 ▸; Nugent *et al.*, 1996 ▸).

The sensitivity and dynamic range, which indicates the extent to which large differences in density can be observed correctly, of phase imaging are related in a trade-off, and the properties of each method differ significantly, as shown in Table 3 ▸. The sensitivity of the crystal-based X-ray interferometry is the highest, and the dynamic ranges of DEI and the propagation-based phase-contrast imaging are the largest (Pagot *et al.*, 2005 ▸; Yoneyama *et al.*, 2008 ▸; Sun *et al.*, 2013 ▸; Yoneyama, Baba, Hyodo *et al.*, 2015 ▸). Therefore, selecting an optimized phase-imaging method depending upon the sample density distribution is very important when performing a fine observation, as it takes advantage of the high sensitivity of phase imaging. As shown in Fig. 5 ▸, the SAGA Light Source is one of the few SR facilities in the world where all methods are available, *i.e.* grating-based interferometry for large-field and high-speed phase imaging using quasi-monochromatic SR, DEI and crystal-based X-ray interferometry for large-field and fine phase imaging using monochromatic SR, and propagation-based phase-contrast imaging for high-spatial-resolution phase imaging.

#### 3.3.1. Grating-based X-ray interferometry

**Figure 10:**
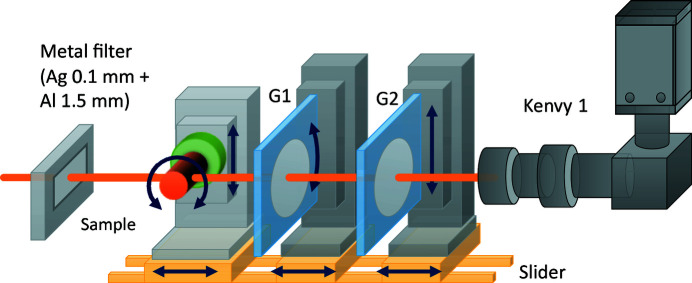
Schematic view of the phase imaging system using an X-ray grating-based interferometer. Quasi-monochromatic SR obtained by a metal filter in the beam path is used for fast phase imaging.

High-speed phase imaging combined with quasi-monochromatic SR obtained by metal filters and a grating-based X-ray interferometer can be performed in the optical hutch (shown as the orange box in Fig. 5 ▸). The imaging system consists of a water-cooled metal filter, sample rotational and positioning tables, a phase grating (G1) and its positioning tables, an absorption grating (G2) and its positioning tables, and Kenvy 1, as shown in Fig. 10 ▸. The positioning tables of the sample, G1 and G2 are mounted on an optical rail laid on the optical base, and each distance can be easily adjusted by sliding the tables on the rails.

The phase grating (G1) has a period of 4.73 µm and is made of Au with a height of 1.9 µm, which shifts the phase of 20 keV X-rays by 180°; the absorption grating (G2) has a period of 4.8 µm and is made of Au with a height of 150 µm to sufficiently absorb X-rays of 20 keV or higher. The calculated Talbot interference distance is 180 mm for 20 keV X-rays. Each grating was fabricated on the center of a 5′′ (125 mm) Si wafer measuring 50 mm × 50 mm, which is sufficiently large for the beam size (20 mm × 3 mm). A fringe-scanning method (Bruning *et al.*, 1974 ▸), which calculates the phase shift from multiple differential interference images obtained at different phase shifts, is used to detect the quantitative differential phase shifts caused by the sample. To shift the phase, G2 is moved in the *Z* direction using the positioning table, and the interference images at each phase difference are acquired. Note that the CT measurement is performed using a continuous scanning method (Kibayashi *et al.*, 2012 ▸; Yashiro *et al.*, 2018 ▸) in which G2 is moved in the *Z* direction, and the sample is simultaneously rotated to shorten the measurement time.

**Figure 11:**
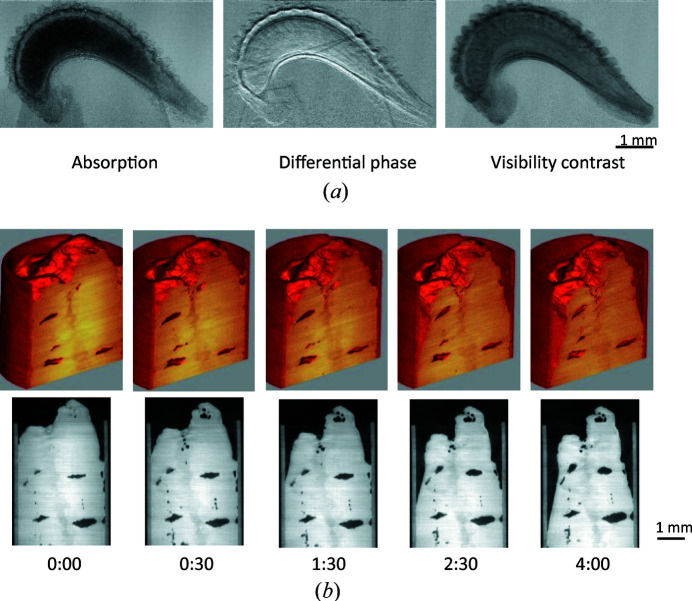
(*a*) 2D images of calendula seed and (*b*) time-resolved 3D images (upper) and sectional images (lower) of cooked rice over 4 h by grating-based interferometry.

Fig. 11 ▸(*a*) shows 2D absorption-, differential phase- and visibility-contrast images of a calendula seed. The exposure time for each interference image was 0.5 s and the number of fringe scans was 10. The metal filters were 1.5 mm-thick Al and 0.5 mm-thick Ag, so the peak energy was 22 keV. The magnification of the object lens of Kenvy 1 is 2, the field of view is 8.3 mm × 7.0 mm, and the pixel size is 3.2 µm^2^. The numerical aperture of the ×2 lens is very small (0.055), so a long exposure time was required. These results show that the contrasts of each image differ from one another. The absorption-contrast image shows the dense internal regions and the visibility-contrast image shows the detailed internal structure, while the differential phase-contrast image shows the fine surface structure.

Fig. 11 ▸(*b*) shows the time-resolved 3D phase-contrast images of cooked rice that were acquired over the course of 4 h. The exposure time of each interference image was 0.1 s, the number of fringe scans was 5, the number of projections was 200 and the total measurement time was 100 s because continuous scanning was used. The images show that the rice dries out and gradually shrinks while the bubbles inside gradually increase in size. The density decreased by about 20% during the measurement.

#### 3.3.2. Crystal-based X-ray interferometry

**Figure 12:**
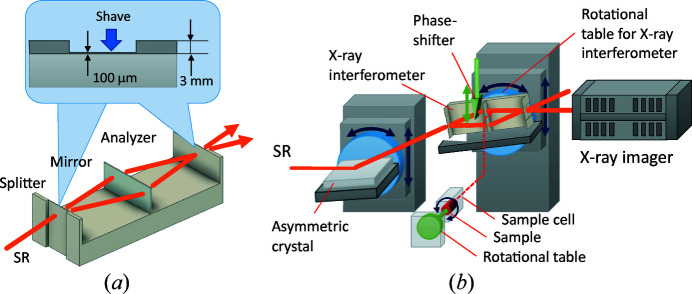
(*a*) Schematic views of the monolithic XI with thin splitter and analyzer and (*b*) imaging system using XI.

Crystal-based X-ray interferometry detects phase shifts using a crystal X-ray interferometer (XI) cut from a large perfect-crystal ingot. The conventional monolithic triple Laue case (LLL) XI (called the Bonse–Hart type) has three parallel crystal wafers, as shown in Fig. 12 ▸(*a*), and it functions in the same way as a Mach–Zehnder interferometer in the visible-light region. In particular, incident X-rays are split by the first wafer (splitter, S), reflected by the second wafer (mirror, M) and combined at the third wafer (analyzer, A) to form two interference X-ray beams. The phase shift caused by the sample placed in the object beam path is converted into intensity changes in the interference beams due to wave superposition, which are detected directly; hence, the sensitivity of this method is the highest among the phase imaging methods.

A phase-contrast X-ray imaging system equipped with a two-crystal XI consisting of two crystal blocks, each having two crystal wafers, has been developed for non-destructive 3D observation of large samples at the Photon Factory in Japan (Yoneyama *et al.*, 2013 ▸), and researchers have taken advantage of its high sensitivity to carry out various observations in biomedicine (Yoneyama *et al.*, 2006 ▸; Noda-Saita *et al.*, 2006 ▸; Takeda *et al.*, 2012 ▸; Yamada *et al.*, 2012 ▸; Kishimoto *et al.*, 2016 ▸; Thet Thet *et al.*, 2018 ▸; Kanahashi *et al.*, 2019 ▸), environmental science (Takeya, 2006 ▸) and industrial science (Takeya *et al.*, 2012 ▸; Mimachi *et al.*, 2015 ▸; Takamatsu *et al.*, 2018 ▸, 2020 ▸). The advantages of two-crystal XI are its large field of view (>30 mm^2^) and large sample space; on the other hand, operation of such an interferometer requires extremely high rotational stability (sub-nrad order) between the crystal blocks (Becker & Bonse, 1974 ▸). Since the beam size of BL07 is small (20 mm × 20 mm), we installed a monolithic XI that uses Si (220) diffraction (Sharan Instruments, Japan). The absorption of one 1 mm-thick crystal wafer is about 25% for 17.8 keV SR, and the intensity of the interference beams is decreased to less than 5% of the incident SR intensity. To reduce the wafer’s absorption, the thicknesses of the splitter (S) and analyzer (A) have been reduced from 3000 to 100 µm by shaving, as shown in Fig. 12 ▸(*a*). The coherence length of the SR is a few micrometres, and the differences in the spacing between the crystal wafers (S–M and M–A) have to be kept equal or less for good interference; thus, the incident side of S and outgoing side of A are shaved. Note that the thickness of M is 1 mm.

Fig. 12 ▸(*b*) shows a schematic view of the phase-contrast imaging system using XI with thinned wafers (shown as the yellow box in Fig. 5 ▸). The system consists of an asymmetric crystal (to expand the SR vertically), the XI and its positioning system, the sample holder and its positioning system, and the X-ray imager (Zyla or VHR). The height of the incident monochromatic SR is less than 5 mm, even at experimental hutch 2, so we installed an asymmetric crystal with an asymmetry angle of 5° to expand the beam in the vertical direction. One of the interference beams generated by the XI is detected by the X-ray imager. Since both the asymmetric crystal and the interferometer require sub-µrad angular stability to perform fine observations, they are mounted on high-precision goniometers to precisely control the incidence angle. The entire imaging system is covered with a curtain to shield the airflow in the experimental hutch, and the temperature fluctuation around the system is no more than 0.5°C during the course of 12 h. Note that the monolithic X-ray interferometer is affected by sample heat because the distance between the sample and the crystal wafer is short. Therefore, observations are limited to samples at the same temperature level as room temperature.

If the spacing of the interference fringes is narrower than the spatial resolution of the X-ray imager, the fringes cannot be detected accurately, and phase unwrapping cannot be performed correctly [phase-unwrapping error is the inability to detect whether the phase jump is 2π*n* or 2π(*n* + 1)]. To eliminate this error, the sample is placed in a liquid such as water, saline solution or ethyl­ene glycol, whichever has the closest density to that of the sample, in order to widen the fringe spacing at the sample’s boundary. In the CT measurement depicted in Fig. 12 ▸(*b*), the sample is rotated around the horizontal axis in the liquid cell. As with grating-based interferometry, the fringe scanning method is used for quantitatively measuring phase shifts; therefore, an acrylic wedge is inserted vertically into the interferometer’s optical path as a phase shifter, and the phase is shifted by moving the wedge up and down.

**Figure 13:**
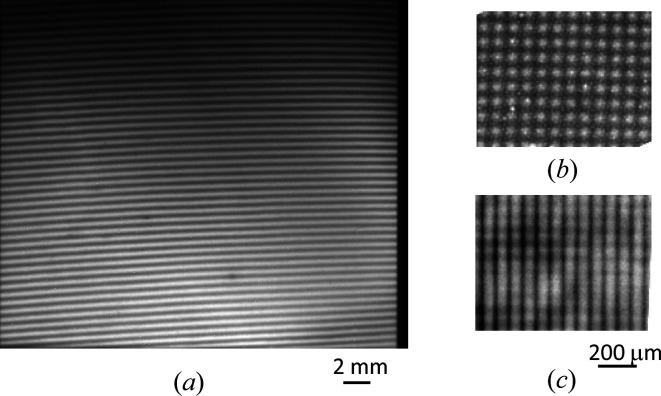
(*a*) X-ray interference image acquired by the XI with thin S and A wafers. Interference images of Au mesh (400 line-space inch^−1^) acquired by XI with thinned wafers (*b*) and conventional XI (1 mm-thick wafers) (*c*). Image (*c*) is blurred in the diffraction direction (vertical direction) by the Borrmann fan effect.

Fabrication of a monolithic XI with the A-wafer center thinned by etching and an interference image of a plastic ball without blurring was reported (Hirano & Momose, 1999 ▸). However, no operational result of XI with thinned S and A wafers has been reported in the literature, so we first tested the performance (intensity, uniformity and visibility) of the interference pattern using monochromatic 17.8 keV SR. Fig. 13 ▸(*a*) shows an X-ray interference pattern obtained using the Zyla with a 1 s exposure time. The size of the pattern (field of view) is about 15 mm × 12 mm, which is large enough to observe a small sample of animal organs. The spatial distribution of visibility is almost uniform; average visibility is 40%, which is lower than that of a conventional monolithic XI (∼80%). Note that the intensity at the top of the pattern is lower because of the lower incident SR intensity.

The advantage of the XI with thinned S and A wafers is not only the SR’s high throughput but also its lack of image blur from the Borrmann fan effect. According to the dynamical theory of X-ray diffraction (Batterman & Cole, 1964 ▸), the X-ray beam path inside the crystal wafer is very sensitive to deviations from the Bragg angle, and even a small angular change causes a large positional shift of the outgoing beam. Any slight angular change caused by sample refraction changes the beam path in the A wafer significantly, resulting in a blurred interference image (the Borrmann fan effect). Thus, the XI with a thin A wafer makes it possible to perform high-spatial-resolution observations. Figs. 13 ▸(*b*) and 13 ▸(*c*) compare the interference patterns of an Au mesh (400 line-space inch^−1^) obtained using the XI with thinned S and A wafers and a conventional XI (1 mm-thick wafers). The patterns were recorded under the same conditions, *i.e.* X-ray energy of 17.8 keV and Zyla imager, except for (*c*), which was obtained at the beamline BL16B2 of SPring-8. The mesh is clearly visible in the vertical and horizontal directions in (*b*); in contrast, the mesh is blurred in the vertical direction (parallel to the diffraction plane) in (*c*). The blurring caused by the Borrmann fan effect is given approximately by the product of the crystal thickness and the Bragg angle, and it is calculated to be 35 µm for the 100 µm wafer and 350 µm for the 1 mm wafer. Therefore, the blurring in (*c*) is mainly caused by the Borrmann fan effect.

**Figure 14:**
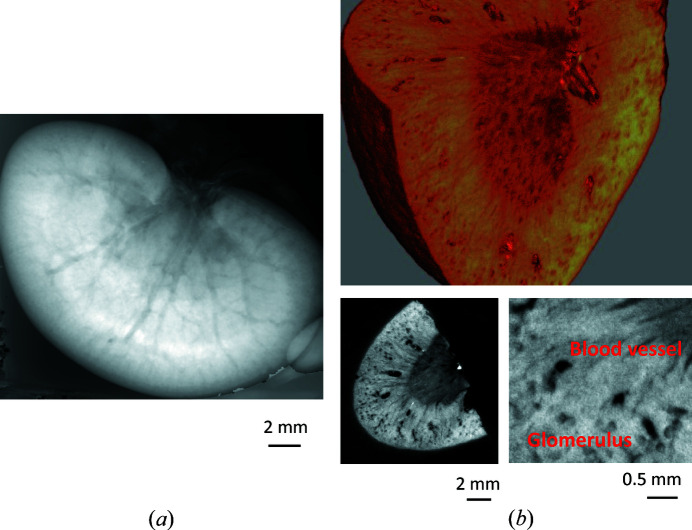
(*a*) Phase map and (*b*) 3D image (top), whole sectional image (bottom left) and magnified sectional image (bottom right) of mouse kidney by crystal-based X-ray interferometry.

Fig. 14 ▸(*a*) shows the phase maps (spatial distributions of phase shift) of a mouse kidney obtained using the XI with thinned S and A wafers. The SR energy was 17.8 keV, the exposure time for acquiring each interference image was 10 s and a six-step fringe scanning method was used. The Zyla was used as the X-ray imager, so the pixel size is 6.5 µm. Owing to the high sensitivity of the crystal-based X-ray interferometry, the microvasculature inside the kidney could be visualized in detail without using any contrast agent.

Fig. 14 ▸(*b*) shows the 3D and sectional images of a piece of mouse kidney obtained by phase-contrast CT. The X-ray energy was 19.0 keV, the exposure time for one interference image was 5 s and the phase map of each projection angle was acquired using a three-step fringe scanning method. The number of projections was 500 over 360° and the total measurement time was about 3 h. Detailed structures of blood vessels, the cortex and glomeruli inside the kidney are clearly visible. The density resolution calculated from the standard deviation in the background area is 1.0 mg cm^−3^, which is about two times larger than that of the phase imaging system at the Photon Factory (Yoneyama *et al.*, 2013 ▸). The deterioration in resolution may be due to the low SR intensity and the low visibility of the interference image. A blood vessel with a diameter of 30 µm is visualized in the enlarged image (bottom right); the spatial resolution is estimated to be finer than 30 µm.

#### 3.3.3. Propagation-based phase-contrast imaging

**Figure 15:**
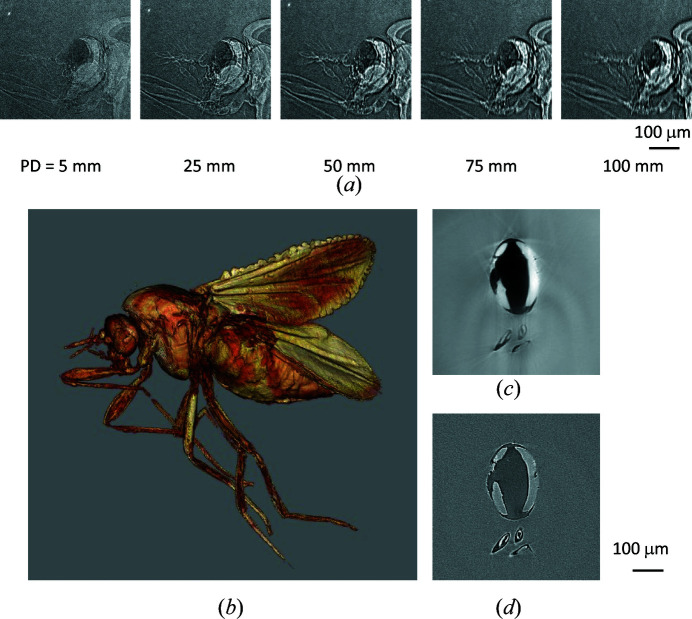
(*a*) Projection image of an insect (Ceratopogonidae) in amber obtained by propagation-based phase-contrast imaging. Edges are enhanced by increasing the PD. (*b*) 3D image and (*c*) cross-sectional image near the abdomen of an insect (Ceratopogonidae) in amber. S/N is 10× higher than that of an absorption-contrast image (*d*) of the same slice.

The propagation-based phase-contrast imaging detects phase shifts by using Fresnel diffraction and does not require any additional X-ray optical components. Therefore, the method is suitable for micro-phase-contrast CT and can be performed by combining SR monochromated using the Ge DCM and Kenvy 2 (shown as the red box in Fig. 5 ▸). The instrumentation configuration is the same as the one used to make Fig. 6 ▸, except that Kenvy 2 is separated from the sample. Fig. 15 ▸(*a*) shows projection images acquired at different PDs of an insect (Ceratopogonidae) trapped in amber. The edges of the insect become more pronounced and the detailed structures can be visualized by increasing the PD. On the other hand, the image becomes blurred when the PD exceeds 50 mm.

Figs. 15 ▸(*b*) and 15 ▸(*c*) show a 3D image and a cross-sectional image near the abdomen of the same sample. The sectional image was reconstructed by conventional filtered back-projection after phase retrieval using an algorithm for a single distance (Weitkamp *et al.*, 2011 ▸). Each projection image was acquired at a PD of 20 mm and 10 keV SR. The exposure time was 2 s, and the number of projections was 1000 over 360°. The images clearly illustrate the detailed structures inside the insect’s body and its wings. Fig. 15 ▸(*d*) shows a sectional image reconstructed by conventional filtered back-projection without phase retrieval for the same CT data (absorption-contrast image). The signal-to-noise ratio (S/N) computed using the CT value in the abdomen as the signal and fluctuations (standard deviation) in the background region as noise in (*c*) is about 10× higher than that in (*c*). Note that (*d*) was measured at a single distance, so the density is not quantifiable as the precise values for the index of refraction are unknown.

### 3.4. Low-temperature CT

**Figure 16:**
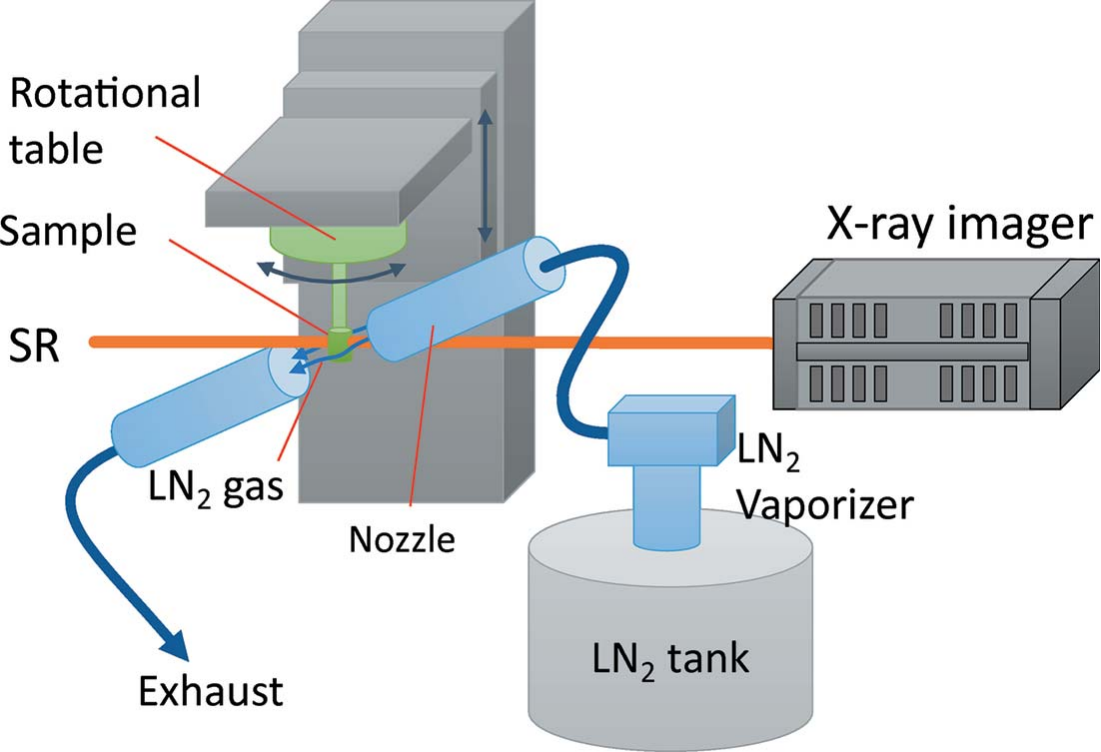
Schematic view of the low-temperature CT system. The sample is kept at low temperature by nitro­gen gas flow.

3D observations made at low temperature of samples such as clathrate hydrates and frozen foods can be performed using a combination of SR monochromated by the Si DCM and a sample-cooling (cryogenic) system in experimental hutch 2, as shown in Fig. 16 ▸ (shown as the blue box in Fig. 5 ▸). The custom-made cryogenic system consists of a liquid-nitro­gen evaporator, a low-temperature dry-nitro­gen-gas blowing nozzle 15 mm in diameter, a liquid-nitro­gen tank and a controller. The sample is cooled by flowing evaporated dry nitro­gen gas at 123 K from a nozzle. To prevent condensation of ice on the sample, the gas flow is exhausted using an aspirator from the sample’s opposite side. The sample positioning system for the CT measurements consists of remotely controlled rotation and vertical tables.

**Figure 17:**
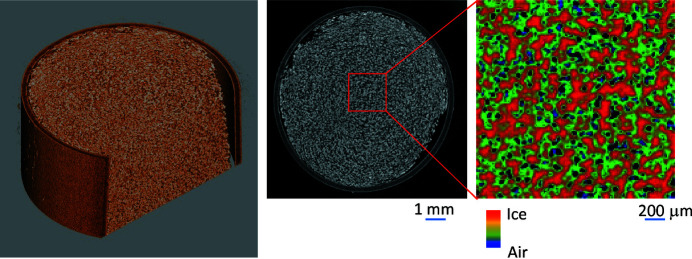
3D (left), whole sectional (center) and magnified sectional (right) images of ice cream observed at low temperature. Air bubbles and ice pieces are clearly visualized.

Fig. 17 ▸ shows a 3D image (left), whole sectional image (center) and magnified sectional image (right) of ice cream confined in a polypropyl­ene tube with a 2 mm diameter. The X-ray energy was 15 keV, the exposure time was 1 s, the number of projections was 1000 over 360° and the total measurement time was 1000 s. The Zyla was used as the X-ray imager, so the pixel size is 6.5 µm. Low-temperature nitro­gen gas was blown continuously over the sample to keep it cold during the measurement. The magnified sectional image reveals air bubbles and ice, as was found in previous reports (Pinzer *et al.*, 2012 ▸; Dalen *et al.*, 2016 ▸). The density resolution is estimated to be 0.10 g cm^−3^ using the standard deviation of the background region and the polypropyl­ene tube’s CT value. The spatial resolution is higher than 20 µm, because the images show air bubbles that are about 20 µm in diameter.

### 3.5. Time-resolved topography

X-ray topography is a high-sensitivity method for detecting dislocations and defects in crystals. It is widely used to evaluate crystalline materials such as silicon, silicon carbide (SiC), diamond and gallium nitride. Since the size of a typical dislocation, such as a screw dislocation, is very small (a few micrometres) compared with a crystal wafer or semiconductor device, a detecting device with high spatial resolution and a large field of view is indispensable. X-ray films or nuclear emulsion detectors can be used for this purpose, but their measurements are limited to static observations. It has been revealed that stacking faults expand in SiC power devices when they are in operation, which may cause severe deterioration of the device’s electrical characteristics. Furthermore, stacking faults are much larger than screw dislocations; thus, they can be detected dynamically with an X-ray imager such as the Zyla. We have developed a time-resolved X-ray topography system to observe the expansion of stacking faults inside operating semiconductor devices by using a combination of SR monochromated using the Si DCM and the Zyla (shown as the cyan box in Fig. 5 ▸). Note that the white SR at BL07 is very strong, and its use might have accelerated the expansion of stacking faults and dislocations; thus, monochromatic SR was used.

**Figure 18:**
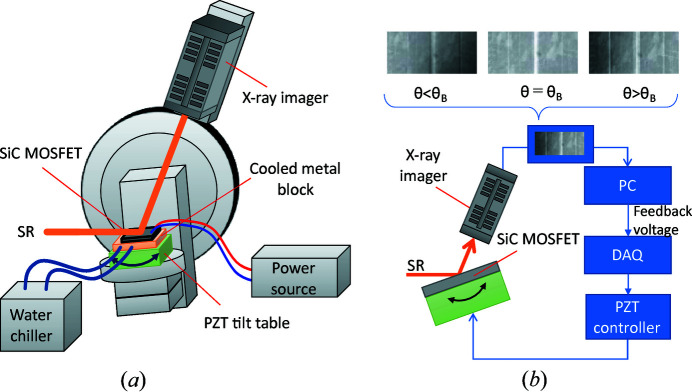
(*a*) Schematic view of the time-resolved topography system and (*b*) feedback system for stabilizing the X-ray’s incident angle using observed topography.

Fig. 18 ▸(*a*) is a schematic view of the topography system, which mainly consists of an X-ray diffractometer with a 2θ arm, a sample positioner, an X-ray imager (Zyla), a sample-cooling system and a power supply for operating the device being tested. The monochromatic SR selected by the Si DCM is formed by a four-blade slit (not shown) and irradiates the sample. The X-rays diffracted by the sample are detected by the Zyla attached to the 2θ arm. The device is mounted directly on the bonded copper substrate by soldering and set on a sample holder (Cu block), which is water-cooled to 12°C using a high-precision chiller.

We also developed a feedback system to stabilize the X-ray’s incident angle to the sample. The system consists of a tilt table driven by a piezoelectric translator (PZT), a PZT controller, digital–analog data acquisition (DAQ) and a control PC, as shown in Fig. 18 ▸(*b*). If the incident angle drifts because of the temperature change caused by operation of the device, the intensities on the left or right sides of the topographic image increase, as shown in Fig. 18 ▸(*b*). The feedback system detects the intensity difference, calculates the compensation voltage of the PZT needed to make the intensity the same, and changes the voltage applied to the PZT via the DAQ to correct the incident angle.

The tilt table’s angular range is ±0.15°, which is sufficiently larger than the angular drift caused by the temperature change (∼100°C). The positioning accuracy (angular resolution) of the table is less than 1/100 µrad, and the X-ray’s incident angle can be controlled with sufficient accuracy to satisfy the Bragg diffraction condition of the SiC. The exposure time needed for obtaining a topographic image is usually 1–10 s, so the operating frequency of the feedback is less than 1 Hz; however, the thermal drift rotation is very slow, from several minutes to several hours, so that it can be suppressed sufficiently even at a slow operating frequency.

**Figure 19:**
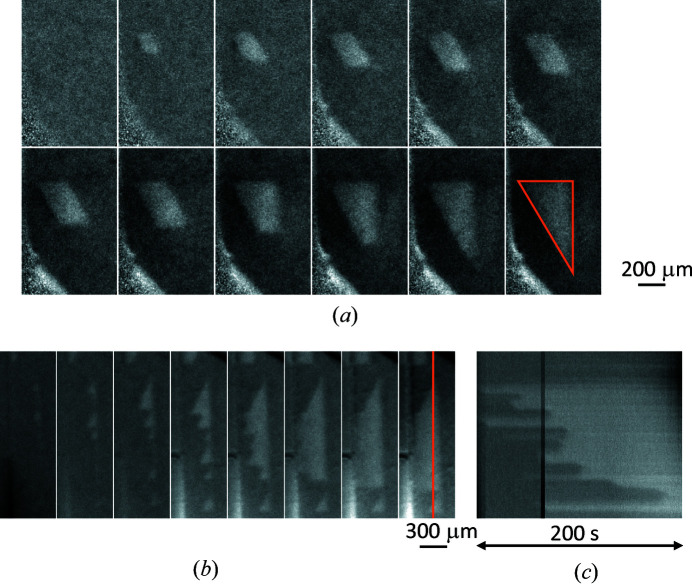
(*a*) Topographic images of a slowly expanding stacking fault (taken at 2 min intervals) and (*b*) a quickly expanding stacking fault (taken at 25 s intervals), and (*c*) time chart of diffracted X-ray intensity of the orange line in (*b*).

Time-resolved topography of a SiC metal-oxide-semiconductor field-effect transistor (MOSFET) was performed using monochromatic 10 keV SR. SiC (0 −2 2 10) diffraction was used for the observation because the stacking faults in the SiC MOSFET appear as bands (Konishi *et al.*, 2021 ▸). The calculated incidence and diffraction angles were 17.4° and 83.6° for 4H-SiC with 4° off-axis (0001). Fig. 19 ▸(*a*) shows time-resolved topographic images of the device in operation. The exposure time was 10 s, but the interval of each displayed image was 2 min because of its slow expansion rate. The growth of a stacking fault over the course of 20 min is clearly visible. Fig. 19 ▸(*b*) shows time-resolved topographic images of the same device taken with a 25 s interval. The expansion was faster than that of the stacking fault in the upper image and saturated within 2 min. Also, it rapidly extended downwards after saturation. Fig. 19 ▸(*c*) shows a time chart of the diffracted X-ray intensity of the orange line in the topography. The chart indicates that the origin of the stacking fault was not one point but rather several points and that the faults spread gradually before finally merging. These results show that time-resolved topography makes it possible to quantitatively evaluate how stacking faults expand in time and at what speed.

## 4. Conclusion

We have been developing various advanced imaging methods and systems for non-destructive observations of various samples so that researchers in fields ranging from biomedicine to functional materials can take advantage of the brilliant SR emitted from the superconducting wigglers at BL07 of the SAGA Light Source. By combining white, quasi-monochromatic and monochromatic SR, fast and low-dose 3D micro-CT observations and 2D micro-XAFS observations can be performed at the optical hutch. Additionally, numerous large-area phase-contrast X-ray imaging techniques are available, including grating-based X-ray interferometry, DEI, crystal-based X-ray interferometry and propagation-based phase-contrast imaging, can be used to perform high-density-resolution observations of biomedical and organic material samples. Low-temperature samples, such as of frozen foods, can be observed with the low-temperature CT system. Moreover, the time-resolved topography system can be used to analyze quantitatively, *e.g.* the expansion of stacking faults in power devices in operation. The next step in our developments will be to devise and install high-speed SXFM using optimized focusing mirrors (Matsuyama *et al.*, 2016 ▸), high-speed CT combined with a fast X-ray imager and white SR, and a micro-phase-contrast low-temperature CT for bio­medical and industrial applications.

## Figures and Tables

**Table 1:** Main specifications of X-ray imagers at SAGA Light Source

	Manufacturer and name	Type	Field of view (mm)	Pixel size (µm)	Pixel number	Phosphor
1	SAGA-LS, Kenvy 1	Lens coupling	3.3 × 2.8 for ×5 lens	1.3 for ×5 lens	2560 × 2160	Lu_3_Al_5_O_12_:Ce (LuAG)
			1.6 × 1.4 for ×10 lens	0.65 for ×10 lens		
2	SAGA-LS, Kenvy 2	Lens coupling	2.6 × 2.6 for ×5 lens	1.3 for ×5 lens	2048 × 2048	CsI:Tl (CsI)
			1.3 × 1.3 for ×10 lens	0.65 for ×10 lens		
3	Photonic Science, VHR	Fiber coupling	50 × 35	12.5	4008 × 2650	Gd_2_O_2_S:Tb (GOS)
4	Andor, Zyla 5.5 HF	Fiber coupling	16 × 13	6.5	2560 × 2160	CsI:Tl (CsI)

**Table 2:** Main specifications of X-ray imaging methods at SAGA Light Source

Imaging method	X-ray optics	Scan time	Spatial resolution	Detector	Application example
Micro-CT	Ge DCM	1 h	2 µm	Kenvy 2	Food, wood, microfossils
	Metal filter	100 s	2 µm	Kenvy 1	Wood, microfossils
Micro 2D XAFS	Ge DCM	1 h	5 µm	Kenvy 2	Fe, Cu powder
Scanning X-ray fluorescence microscopy (SXFM)	–	1 h for 200 µm × 200 µm	1 µm	PIN, SDD	Elemental mapping of seeds, microfossils and ash
Grating-based interferometry	Metal filter	5 min for CT	100 µm	Kenvy 1	Food
Diffraction-enhanced imaging (DEI)	Si DCM	1 h for CT	30 µm	Zyla, VHR	Biomedical and organic samples
Crystal-based interferometry	Si DCM	3 h for CT	30 µm	Zyla, VHR	Biomedical and organic samples
Propagation-based imaging	Ge DCM	1 h for CT	2 µm	Kenvy 2	Food, wood, microfossils
Low-temperature CT	Si DCM	15 min	30 µm	Zyla, VHR	Food, clathrate hydrates
Time-resolved topography	Si DCM		30 µm	Zyla, VHR	SiC power devices

**Table 3:** Properties of phase-detecting methods available at SAGA Light Source

Method	Phase-detecting device	Sensitivity	Dynamic range	Scan time for CT	Spatial resolution
Grating-based X-ray interferometry	X-ray gratings	Low	Middle	2 h for mono SR	∼10 µm
				5 min for white SR	
Crystal-based X-ray interferometry	Crystal X-ray interferometer	High	Narrow	∼2 h	∼30 µm
Diffraction enhanced imaging	Analyzer crystal (single crystal)	Middle	Middle	<30 min	∼10 µm
Propagation-based imaging	–	Low	Wide	1 h	∼3 µm
